# Designing a Multi-Epitope Vaccine Against MPXV and HIV Based on an Immunoinformatic Approach

**DOI:** 10.3390/ijms26136313

**Published:** 2025-06-30

**Authors:** Ding Tang, Siwen Wu, Youchun Wang, Weijin Huang

**Affiliations:** 1 Division of HIV/AIDS and Sex-Transmitted Virus Vaccines, Institute for Biological Product Control, National Institutes for Food and Drug Control (NIFDC), Beijing 102629, China; 2 Chinese Academy of Medical Sciences & Peking Union Medical College, Beijing 100006, China; 3 Institute of Medical Biology, Chinese Academy of Medical Sciences, Kunming 650118, China

**Keywords:** MPXV, HIV, immunoinformatic, multi-epitope vaccine

## Abstract

In the current global health environment, the spread of the monkeypox virus (MPXV) and the persistent threat of human immunodeficiency virus (HIV) have become critical public health challenges. Since 2022, MPXV has rapidly disseminated worldwide, and nearly half of MPXV-infected individuals are co-infected with HIV. This complex situation calls for innovative preventive strategies. In this study, an innovative multi-epitope vaccine was designed using bioinformatics and immunoinformatic approaches. Ten HIV proteins and nine MPXV proteins were used to predict potential epitopes. Non-allergenic, highly antigenic, IFN-γ-inducible, and non-toxic epitopes were selected to construct the multi-epitope vaccine. It was found that the designed vaccine construct was highly antigenic, soluble, and had acceptable physicochemical properties. Based on molecular docking and molecular dynamics simulation (MDs) analyses, the vaccine construct demonstrated stable and robust interactions with Toll-like receptors (TLR2, TLR3, and TLR4). Although no actual animal experiments have been conducted to evaluate the vaccine’s effectiveness, immune simulations showed that the vaccine could elicit potent humoral and cell-mediated immune responses. Overall, this study provides a promising vaccine candidate against MPXV and HIV co-infection and emphasizes innovative strategies to interrupt the international transmission of these two viruses.

## 1. Introduction

By 30 November 2024, the World Health Organization (WHO) had received reports of 117,663 laboratory-confirmed cases of MPXV infection, including 263 fatalities [1]. Before 2022, MPXV was disseminated in a few African countries and was not considered an epidemic. However, as the MPXV spread globally, on 22 November 2024, the WHO again declared the mpox outbreak to be a ‘public health emergency of international concern’ (PHEIC) [2]. Mpox is caused by the MPXV, an enveloped double-stranded DNA virus belonging to the *Orthopoxvirus* genus [3]. It is classified into two genetic lineages: the West African lineage (fatality rate less than 1%) and the Central African lineage (fatality rate: 11%) [4]. MPXV can be spread from person to person through respiratory droplets, sexual contact, and characteristic skin lesions [5]. In addition, there is evidence that MPXV can infect the fetus through vertical transmission or cause miscarriage [6]. Vaccines are a cost-effective way to prevent infection by pathogens. The WHO recommends three smallpox vaccines for use in high-risk exposed populations, including MVA-BN (non-replicating), LC16 (minimal replication), and ACAM2000 (vaccine-based replication) [7]. Unfortunately, these vaccines were not developed exclusively for MPXV and have some limitations, including causing serious adverse events (e.g., progressive vaccinia, myocarditis, death) or limited availability of the vaccine [8,9]. In addition, it was found that antibodies induced by the smallpox vaccine were inefficient in neutralizing MPXV [10,11]. Therefore, it is essential to develop specific MPXV vaccines to address potential future outbreaks of mpox.

Although new testing technologies, medications, and improved sanitation have reduced the incidence of infections and deaths associated with infections, Acquired Immune Deficiency Syndrome (AIDS) persists as a major global public health challenge [12,13]. According to UNAIDS estimates, 88.4 million people worldwide have been diagnosed with AIDS since the beginning of the epidemic, and 42.3 million have died of the disease [14]. During 2023, global health data recorded 1.3 million fresh HIV infections alongside 630,000 AIDS-related fatalities [14]. Currently, Highly Active Antiretroviral Therapy (HAART) is the most successful method of treating AIDS, which slows down HIV progression, thereby greatly increasing life expectancy and improving patients’ living standards. HAART has been shown to decrease AIDS-related mortality by approximately 60%, but it cannot completely cure AIDS patients and is associated with high costs [15]. Therefore, a safe, effective, and inexpensive vaccine remains urgently needed to eradicate AIDS. Unfortunately, there is no successful vaccine against HIV to date, and RV144, perhaps the most promising vaccine, offers only a 31.2% protection rate against HIV [16]. Many HIV vaccine candidates have failed in clinical trials owing to their inability to stimulate the body to produce sufficient immune protection [17,18].

On the other hand, both MPXV and HIV can be transmitted through sexual contact. Specifically, 86.7% of the patients in this mpox outbreak were men who have sex with men, and it was found that 48.2% of the mpox patients were co-infected with HIV [1]. Co-infection with MPXV and HIV can exacerbate illness severity, worsen the prognosis, prolong the convalescence period, and may increase the incidence and lethality associated with subsequent diseases [19,20]. Therefore, the design of innovative vaccines capable of impeding the co-infection of MPXV and HIV holds great significance in curbing the global dissemination of these two viruses. Single vaccines designed to protect against multiple viruses have been shown to be feasible. For example, researcher Cao developed a vaccine that provides protection against both SARS-CoV-2 and influenza virus infections. This vaccine includes immunogens formed by combining the receptor-binding domain of SARS-CoV-2 with a conserved stem of the H7N9 hemagglutinin [21]. An optimal vaccine is expected to focus on the wide range of highly conserved segments of the virus, prompting the activation of B-cells, CD4^+^-T-cells, and CD8^+^-T-cells, along with inducing the secretion of appropriate cytokines such as IFN-γ [22]. Moreover, it should incorporate suitable adjuvants to boost the immune response and initiate an appropriate innate immune reaction [22]. Using bioinformatics tools is a promising approach to design and develop valuable vaccine immunogens, as they can rapidly identify highly immunogenic epitopes used in vaccine design [23,24].

Here, we sought to develop a recombinant protein vaccine containing multiple highly immunogenic epitopes through immunoinformatic methodologies, which has the potential to trigger a robust humoral and cell-mediated immune response to combat MPXV and HIV co-infection. However, due to the limitations of computational predictions, in vitro and in vivo experiments (e.g., protein expression validation, animal experiments) are still needed to validate the efficacy of this vaccine in the future.

## 2. Results

### 2.1. T-Cell Epitopes

The selected cytotoxic T lymphocytes (CTLs) and helper T lymphocytes (HTLs) epitopes are shown in Table 1 and Table 2, respectively. The T-cell epitope activates the cellular immune response to clear virus-infected cells and stimulates the humoral immune response through cytokines. Briefly, the T-cell epitopes were included in the candidate pool based on IEDB scores (<0.5), antigenicity (>0.7), toxicity, and allergenicity. Finally, CTL epitopes with the top immunogenicity score and HTL epitopes with the top IFN-γ prediction score were chosen to construct the multi-epitope vaccine.

### 2.2. Linear B-Cell Epitopes

The predicted linear B lymphocyte (LBL) epitopes were ranked based on the ABCpred prediction score, antigenicity, toxicity, and allergenicity. Epitopes with scores of ≥0.90 were selected for the vaccine construct. Interestingly, some proteins, such as M1R, Tat, Rev, Pro, RT, IN, and Vpu, did not produce excellent, qualified epitopes. Table 3 lists the final selected LBL epitopes.

### 2.3. Population Coverage

The worldwide coverage of the HLA I and HLA II epitopes reached 98.55% and 99.99%, respectively. In addition, more detailed analyses of population coverage were carried out for 16 areas worldwide. All of the regions with high monkeypox prevalence (e.g., the Americas, Africa, and Europe) had a population coverage of greater than 88.30% for HLA I epitopes, except for Central America (only 7.76%), and the lowest population coverage was 99.92% for HLA II epitopes. These data imply that the multi-epitope vaccine offers extensive coverage across global populations. Detailed information is shown in Figure S1 and Table S1.

### 2.4. Multi-Epitope Vaccine Construction

**Figure 1:**
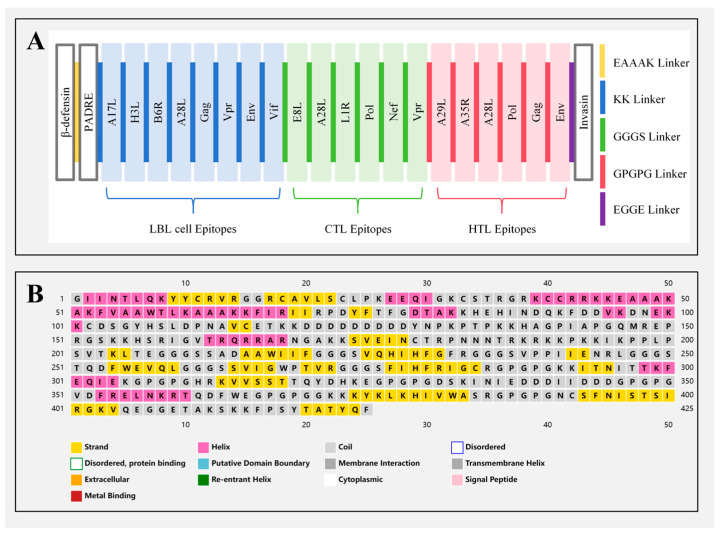
Schematic depiction of the linking strategy and secondary structure of the final multi-epitope vaccine construct. (**A**) The illustration depicts the arrangement of epitopes and the linkers used in the final vaccine construct. (**B**) Secondary structure: The multi-epitope vaccine is composed of 16.94% (72/425) α-helices, 21.41% (91/425) extended strands, and 61.65% (262/425) random coils.

As shown in Figure 1A, six CTL epitopes, six HTL epitopes, and eight LBL epitopes were finally chosen to design the multi-epitope vaccine. To enable each epitope to exert its function separately, the GGGS, GPGPG, and KK linkers were added to separate each epitope to prevent the epitopes from interfering. The adjuvants β-defensin, helper peptide PADRE, and C-terminal invasion sequence of *Yersinia* were also fused to the vaccine via EAAAK and EGGE linkers.

### 2.5. Physicochemical Properties of Multi-Epitope Vaccine

The antigenicity (score: 0.7074) and solubility (score: 0.551) of the multi-vaccine were appropriate. The ProtParam server indicated that there are 425 amino acids in the vaccine, with a molecular weight of 46.63 kDa, theoretical protrusion index (PI) of 9.73, lipid index of 54.16, grand average of hydropathicity (GRAVY) of −0.917, and instability index of 36.01. These results suggested that the vaccine construct folds into a stable structure and exhibits stability across various temperatures. Furthermore, the half-life of the multi-epitope vaccine was 30 h in cultured mammalian reticulocytes, 20 h in yeast, and >10 h in *E. coli.* (Table S2).

### 2.6. Secondary and 3D Structure Analysis of the Vaccine Construct

**Figure 2:**
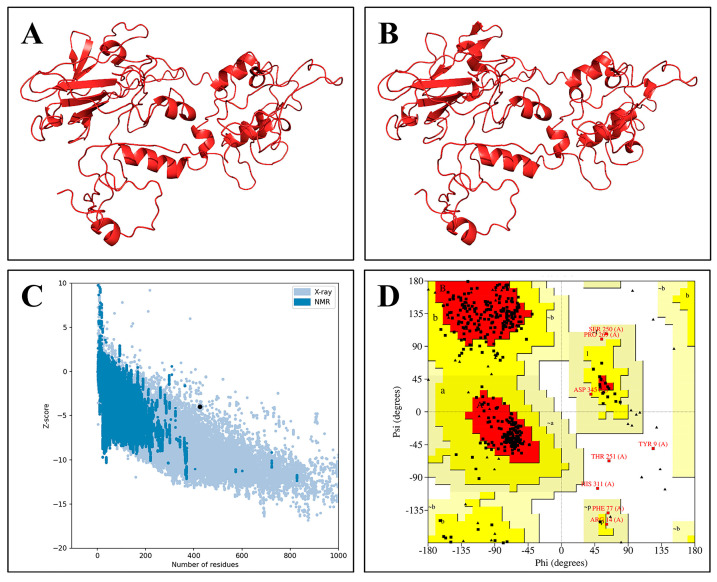
Structural analysis of model 3. (**A**) Initial 3D structure. (**B**) Refined 3D structure. (**C**) Z-score: −3.97. (**D**) Ramachandran plot analysis. Red region: most favorable [A, B, L]; yellow region: additional allowances [a, b, l, p]; pale yellow area: generous allowances [~a, ~b, ~l, ~p].

The vaccine contained 16.94% (72/425) of alpha helices, 21.41% (91/425) of extended strands, and 61.65% (262/425) of random coils (Figure 1B). The I-TASSER server produced five potential 3D models for the vaccine with confidence scores (C-scores) of −1.23, −3.00, −3.57, −3.64, and −3.98, respectively. The model with the highest *C*-score (−1.23), indicating superior structural quality, was chosen for refinement. The GalaxyRefine server produced five refined models. Based on criteria including a higher GDT-HA score, lower RMSD score, lower MolProbity score, and lower Clash score, model 3 was considered the best refined model with a GDT-HA score of 0.9306 (the highest relative value), RMSD score of 0.460, MolProbity score of 2.167, and Clash score of 11.9. Figure 2A,B show the initial and refined 3D structures of the vaccine. The z-score of the ProSA web server was −3.97 for the refined model (Figure 2C). SAVES ERRAT showed that the overall quality factor was 85.6, and VERIFY 3D revealed that 81.4% of the amino acids had scores ≥ 0.1 in the 3D/1D profile. The Ramachandran plot from PROCHECK indicated that 99.1% of the residues were in allowed regions, and only 0.9% were in disallowed regions (Figure 2D). Collectively, these data strongly suggest that this was a high-quality model that could be used for further analysis.

### 2.7. Prediction of Conformational B-Cell Epitopes

According to ElliPro Server results, this vaccine construct contains six discontinuous B-cell epitopes, each scoring above 0.5. These epitopes varied in length, spanning from 3 to 81 amino acids (Figure S2).

### 2.8. Molecular Docking

**Figure 3:**
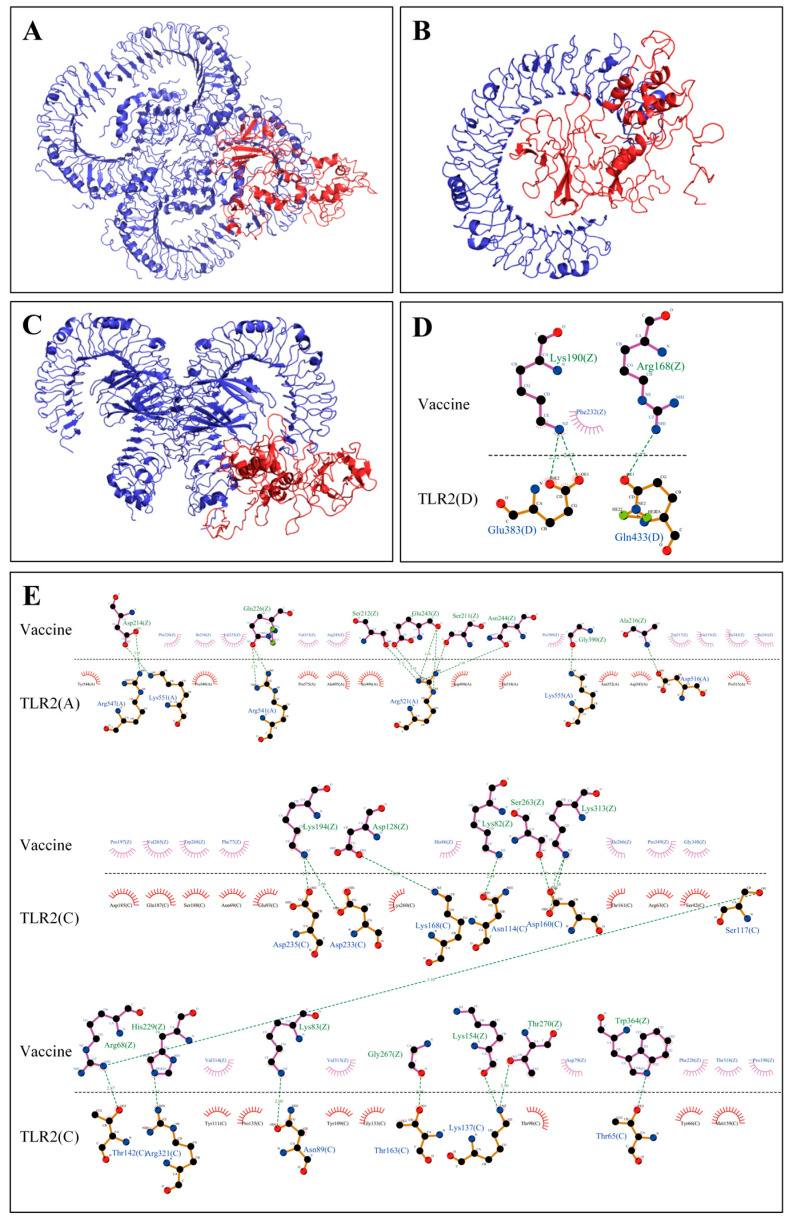
Three-dimensional structural diagram and interactions of molecular docking complexes. (**A**–**C**) Three-dimensional structural diagram of molecular docking of TLR2, TLR3, and TLR4. The vaccine is displayed in red, and TLRs are shown in blue. (**D**,**E**) Interactions between the vaccine and chains D, A, and C of TLR2. Serrated shapes indicate hydrophobic residues; blue, black, and red spheres indicate nitrogen, carbon, and oxygen atoms, respectively; purple solid lines indicate ligand bonds, and brown solid lines indicate non-ligand bonds, green dotted lines represent hydrogen bonds.

**Figure 4:**
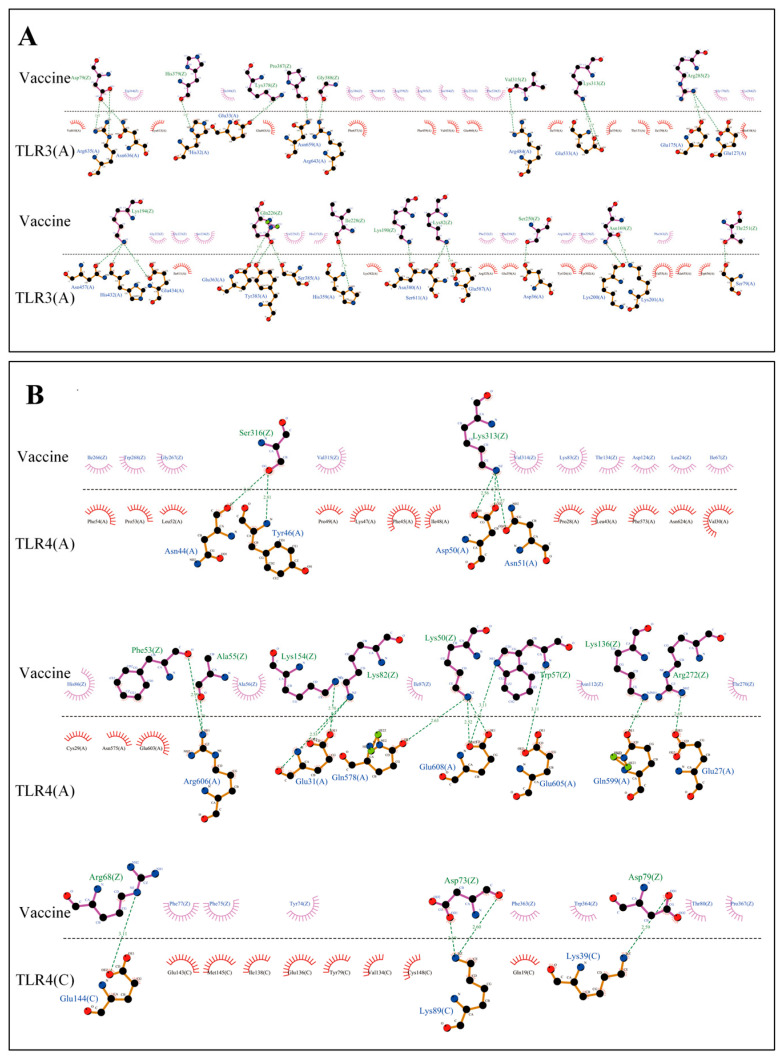
Interactions between the vaccine and TLR3 (**A**)/TLR4 (**B**). Serrated shapes indicate hydrophobic residues; blue, black, and red spheres indicate nitrogen, carbon, and oxygen atoms, respectively; purple solid lines indicate ligand bonds, and brown solid lines indicate non-ligand bonds, green dotted lines represent hydrogen bonds.

After docking with TLR2, TLR3, and TLR4, the server generated 30 models for each docking step. The docking complexes of the vaccine construct with TLR2, TLR3, and TLR4 had the lowest energy scores of −1093.9, −1230.0, and −1187.5, respectively, which were visualized using PYMOL and are shown in Figure 3A–C. The LigPlot (version 2.2.9) results showed that there were 31 hydrogen bonds within the vaccine–TLR2 complex (Figure 3D,E), 26 hydrogen bonds within the vaccine–TLR3 complex (Figure 4A), and 22 hydrogen bonds within the vaccine–TLR4 complex (Figure 4B). These results revealed that the vaccine construct could bind stably to these three TLRs, eliciting a potent immune response. In addition, Tables S3–S5 show the list of residues with the hydrogen bonds of the vaccine with TLR2, TLR3, and TLR4, as well as the bond length.

### 2.9. Molecular Dynamics Simulation

**Figure 5:**
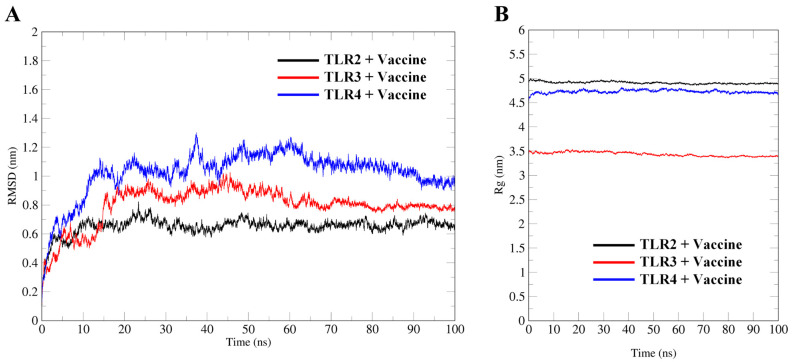
MD analysis of vaccine–TLR complexes. RMSD (**A**) and Rg (**B**) plots of the vaccine and TLR docking complex for a time duration of 100 ns, where RMSD reflects structural stability, and Rg indicates the structural compactness of the complexes.

GROMACS conducted the MDs of the vaccine–TLR complexes for 100 ns. RMSD is an important index for evaluating the structural stability of protein complexes during MDs [25]. At the beginning of the MDs, the RMSD values of the three complexes quickly rose (Figure 5A). The RMSD values of complexes of TLRs with the vaccine peaked at approximately 0.8, 0.9, and 1.3 nm at 24 ns, 26 ns, and 38 ns, respectively. Then, the systems experienced fluctuations within a relatively small range, suggesting the excellent stability of the docking complex. The Rg values of the three complexes fluctuated within a very small range, indicating the good compactness of the tertiary structure (Figure 5B). The TLR2 and TLR4 receptors have relatively large molecular weights and, therefore, have large Rg values.

### 2.10. Immune Simulation of the Multi-Epitope Vaccine

**Figure 6:**
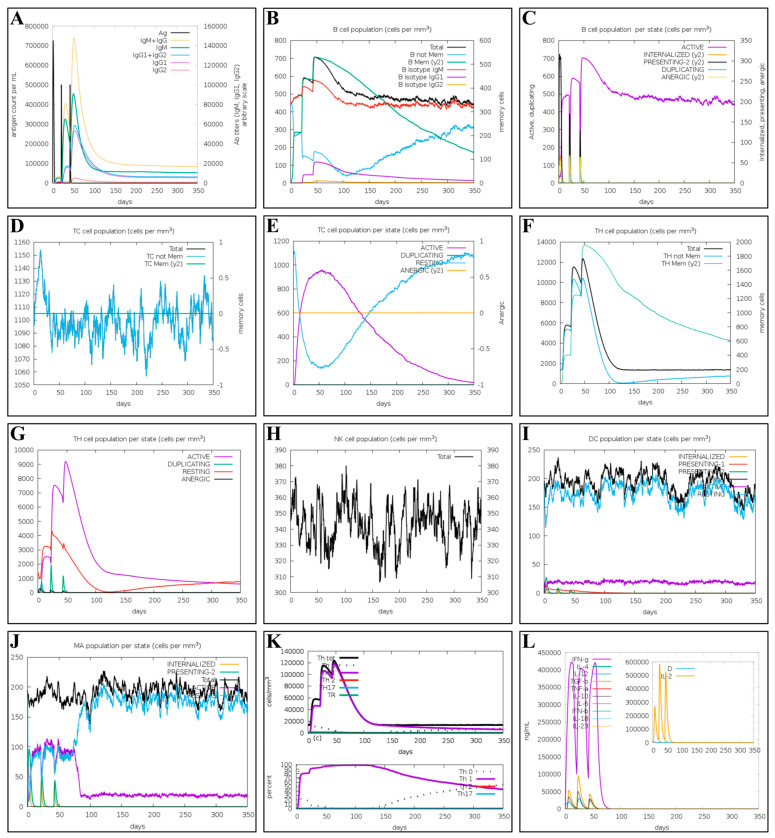
Immune simulation results for the multi-epitope vaccine. (**A**) Immunoglobulin production after immunization. Antibodies were subdivided into isotypes. (**B**) Count of B cells: total count, memory cells, IgM, IgG1, and IgG2 isotypes. (**C**) B cell population stratified by entity state. (**D**) Count of CD8^+^-TC cells. (**E**) Count of CD8^+^-TC cells per entity state. (**F**) Count of CD4^+^-TH cells. (**G**) Count of CD4^+^-TH cells subdivided per entity state. (**H**) Count of NK cells. (**I**) Count of dendritic cells. (**J**) Count of macrophages. (**K**) The numbers and proportions of T helper cell subtypes. (**L**) Concentrations of cytokines. D in the inset plot is a danger signal.

A computational prediction method, rather than actual animal experiments, was used for the preliminary assessment of vaccine efficacy to reduce the cost of trial and error and increase the success rate of vaccine development. Figure 6 shows the results of the multi-epitope vaccine immune simulation. Antibody titres rose rapidly after vaccination and increased significantly after boost immunisation (Figure 6A). There was a marked rise in the number of memory B cells after each immunisation, peaking at 700 cells/mm^3^, followed by a slow decline over the following year (Figure 6B). Moreover, the number of B cells in an activated state remained between 450 and 500 cells/mm^3^ for a prolonged period (Figure 6C). Notably, the number of T-cytotoxic (TC) cells increased gradually, reaching a peak of 1154 cells/mm^3^, whereas memory TC cells stabilised at 1105 cells/mm^3^ (Figure 6D). Meanwhile, the number of activated TC cells gradually increased and peaked at 980 cells/mm^3^ on day 50 (Figure 6E). The T-helper (TH) cell population formed three gradually rising peaks after vaccination (Figure 6F), with the active TH cell population reaching a maximum of 9000 cells/mm^3^ (Figure 6G). The number of natural killer (NK) cells fluctuated up to 380 cells/mm^3^ on day 100 (Figure 6H). In addition, the significant proliferation of dendritic cells and macrophages was observed (Figure 6I,J). It was observed that the number of Th1 cells increased after each immunization, reaching a maximum of 120,000 cells/mm^3^ (Figure 6K). The prediction results indicated that the vaccine elicited high levels of IFN-γ (420,000 ng/mL) and IL-2 (590,000 ng/mL) (Figure 6L).

### 2.11. Codon Optimization and In Silico Cloning

The optimized sequence (1275 nucleotides) had a CAI value of 1.0 and a GC content of 50.73%, indicating strong theoretical potential for high-level expression in *E. coli*. Then, the optimized sequence was cloned into the pET28a (+) vector to generate a recombinant plasmid (Figure S3).

## 3. Discussion

MPXV has been spreading globally since 2022 and has been classified as a PHEIC twice. To date, no vaccine designed specifically for MPXV has been approved. Although smallpox virus vaccines are effective against MPXV, a vaccine specifically targeting MPXV is needed to avoid immune-avoidance mutations [26]. Concerningly, 48.2% of patients with MPXV were co-infected with HIV, resulting in a more severe prognosis and a higher mortality rate [1]. In the face of such a serious and complex public health situation, the swift advancement in bioinformatics, structural biology, and computational tools has led to various data-driven approaches for selecting and constructing biomarkers in vaccine development [27,28]. Multi-epitope vaccines offer a promising and practical approach to address emerging infectious diseases, as they can be rapidly designed and contain a wide range of antigens [29,30]. Compared to traditional modalities, multi-epitope vaccines designed and evaluated through in silico methods exhibit thermodynamic stability, high efficacy, and specificity while generally being quicker to develop at a lower cost [31].

In this research, we designed a novel multi-epitope vaccine aiming to prevent the co-infection of MPXV and HIV. This vaccine was constructed using immunodominant epitopes derived from nine MPXV antigens and the full range of HIV antigens. To enhance the vaccine’s immunogenicity and antigenicity, we integrated the PADRE helper peptide, β-defensin-3, and the C-terminal invasion sequence of Yersinia into the design. After a predictive analysis of the multi-epitope vaccine’s physicochemical properties, we found that this vaccine had an antigenicity score of 0.7074, solubility score of 0.551, instability index of 36.01, aliphatic index of 54.16, and GRAVY value of −0.917, and it is nontoxic and nonallergenic, being comparable to or even superior to previous vaccine designs in this regard [32,33,34,35]. The results indicate that this vaccine construct has good antigenicity, safety, stability, thermal stability, hydrophilicity, and post-expression solubility, making it an excellent vaccine candidate. We predicted and refined the 3D model of the candidate through the I-TASSER and GalaxyRefine servers and then validated the final model using several servers. The final model’s Z-score was −3.97, aligning with values commonly seen in native proteins of a similar size. The ERRAT overall quality factor was 81.4%, indicating that the final 3D model of the vaccine was satisfactory, and the Ramachandran plot showed that 99.1% of the residues were in allowed regions, which proved the high-quality and stability of the final refined model [36,37].

TLRs mediate the recognition and response to pathogen-associated molecular patterns, activate intrinsic immune cells, and induce cytokine expression [38]. TLR2 and TLR4 recognise viral structural proteins and induce the production of pro-inflammatory cytokines, while HIV-mediated dendritic cell activation is dependent on TLR3 [39,40]. We performed the molecular docking and MDs of docking complexes to explore the specific interactions and binding stability of the vaccine construct with TLRs. Molecular docking analysis showed that 31, 26, and 22 hydrogen bonds formed between the vaccine protein and the TLRs, suggesting a strong binding affinity in all three cases. In addition, it demonstrated that the vaccine could activate the human immune system to produce a robust immune response. MDs revealed that the vaccine–TLR complexes were stable under different pressure, temperature, and movement conditions. The RMSD and Rg values only showed minor fluctuations during the MD process, which indicated that the vaccine–TLR complexes were highly stable in the biological environment without apparent conformational changes [41,42].

Although actual animal experiments have not yet been conducted, the results of the immune simulations imply that this vaccine possesses potentially strong immunogenicity. The C-ImmuSim server, based on a location-specific scoring matrix, simulates the immune response of lymphocytes in mammalian immune organs to the multi-epitope vaccine [43,44]. As a potent vaccine can trigger a potent immune response and generate persistent adaptive immunity, we evaluated the immune responses of immune cells to this multi-epitope vaccine using the C-ImmuSim server [45]. The immune simulation results displayed a remarkable increase in the numbers of B cells, T cells, dendritic cells, macrophages, and NK cells after vaccination, comparable to the actual immune response. Following the initial injection, elevated levels of IFN-γ and IL-2 were sustained through subsequent antigen exposures. These data suggested a robust T helper cell response and efficient immunoglobulin production associated with a strong humoral immune response [46,47]. Consistent with findings from prior immunoinformatic-based vaccine development projects, the vaccine conceived in this study is anticipated to provide immunity against MPXV and HIV [45,47,48].

We performed codon optimization using the JCat web server to enhance transcriptional and translational efficiency, followed by in silico cloning into the pET28a (+) vector. The optimized construct demonstrated a CAI of 1.0 and a GC content of 50.73%, which fell within the optimal ranges. These parameters validated the reliability of the construct for efficient expression in the *E. coli* K12 strain.

This vaccine shows promising potential on several fronts. Firstly, selecting nine conserved antigens of MPXV and a full range of HIV antigens for epitope prediction, covering key immunogenic regions of both viruses, fills a gap in the research on preventing MPXV and HIV co-infection. In contrast, most published studies have only been conducted against a single pathogen [33,36,49]. In addition, a combination of multiple adjuvants was used to enhance antigen presentation and fully activate the immune response compared to a single adjuvant (e.g., β-defensin 2 or cholera toxin B) [32,50]. Compared to the single docking analysis of Choudhury et al. [33], which only focused on TLR2, the present study demonstrated that the vaccine strongly interacted with both TLR2/3/4, covering the critical TLR3 pathway in HIV infection, which is more relevant to the needs of immune activation in co-infections. The level of cellular and humoral immunity induced by this vaccine was comparable to that of the dual-virus vaccine of Jiang et al. [35].

Although computational prediction can accelerate vaccine development, computational tools are limited by algorithmic parameters, training datasets, and the complexity of epitope–receptor interactions and may be subject to prediction bias. Therefore, in vivo and in vitro experiments are still needed to validate the effectiveness of vaccine candidates.

## 4. Materials and Methods

**Figure 7:**
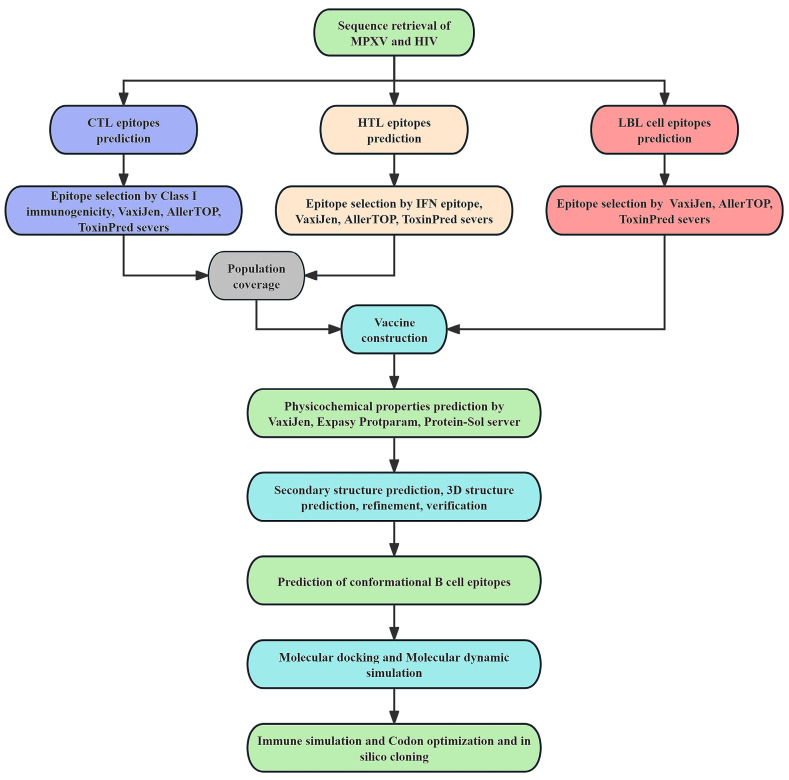
A flowchart depicting the steps taken to develop the multi-epitope vaccine against MPXV and HIV.

Figure 7 illustrates the steps involved in the comprehensive formulation, analysis, and assessment of the multi-epitope vaccine against MPXV and HIV.

### 4.1. Sequence Retrieval

The protein sequences of MPXV (GenBank ID: OP526860.1 (https://www.ncbi.nlm.nih.gov/nuccore/OP526860.1/) (accessed on 4 January 2025) and HIV-1 (GenBank ID: NC_001802.1 (https://www.ncbi.nlm.nih.gov/nuccore/NC_001802.1/) (accessed on 4 January 2025) were retrieved from the NCBI GenBank database (https://www.ncbi.nlm.nih.gov/) (accessed on 4 January 2025). All proteins derived from HIV were collected for further analysis. In addition, carbonic anhydrase (E8L, GenBank ID: UXK31698.1 (https://www.ncbi.nlm.nih.gov/protein/UXK31698.1) (accessed on 4 January 2025), orthopoxvirus A26L/A30L protein (A28L, GenBank ID: UXK31730.1 (https://www.ncbi.nlm.nih.gov/protein/2309195340) (accessed on 4 January 2025), IMV membrane protein L1R (L1R, GenBank ID: UXK31632.1 (https://www.ncbi.nlm.nih.gov/protein/UXK31632.1) (accessed on 4 January 2025), IMV membrane protein L1R (M1R, GenBank ID: UXK31673.1 (https://www.ncbi.nlm.nih.gov/protein/UXK31673.1) (accessed on 4 January 2025), IMV surface fusion protein (A29L, GenBank ID: UXK31731.1 (https://www.ncbi.nlm.nih.gov/protein/2309195341) (accessed on 4 January 2025), EEV glycoprotein (A35R, GenBank ID: UXK31738.1 (https://www.ncbi.nlm.nih.gov/protein/UXK31738.1) (accessed on 4 January 2025), myristoylated protein (A17L, GenBank ID: UXK31721.1 (https://www.ncbi.nlm.nih.gov/protein/2309195331) (accessed on 4 January 2025), IMV heparin binding surface protein (H3L, GenBank ID: UXK31686.1 (https://www.ncbi.nlm.nih.gov/protein/UXK31686.1) (accessed on 4 January 2025), and EEV type-I membrane glycoprotein (B6R, GenBank ID: UXK31758.1 (https://www.ncbi.nlm.nih.gov/protein/UXK31758.1) (accessed on 4 January 2025) were chosen as candidate antigens for MPXV owing to their superior induction of neutralizing antibodies [49,51,52]. Finally, the selected protein sequences were uploaded into prediction tools for epitope identification.

### 4.2. T-Cell Epitope Prediction Analysis

The IEDB MHC I server (https://nextgen-tools.iedb.org/pipeline?tool=tc1) (accessed on 5 January 2025) and Class I immunogenicity server (https://nextgen-tools.iedb.org/pipeline?tool=tc1) (accessed on 5 January 2025) were employed to identify CTL epitopes. Epitopes possessing percentile levels below 0.5 and immunogenicity scores above 0 were chosen for subsequent analysis [35]. HTL epitopes were predicted using the NetMHCIIpan EL 4.1 prediction method in the IEDB MHC II server (https://nextgen-tools.iedb.org/pipeline?tool=tc2) (accessed on 5 January 2025), and the full human leukocyte antigen (HLA) reference set (HLA-DR, HLA-DP, HLA-DQ) was selected as the human MHC alleles. HTL epitopes with a percentile rank below 0.5 were chosen to evaluate the interferon-gamma (IFN-γ) induction ability using the IFN-γ epitope server (https://webs.iiitd.edu.in/raghava/ifnepitope/predict.php) (accessed on 5 January 2025) [53]. Finally, the antigenicity, allergenicity, and toxicity of the qualified CTL and HTL epitopes were separately analysed by VaxiJen v2.0 (threshold value: 0.4) (https://www.ddg-pharmfac.net/vaxijen/VaxiJen/VaxiJen.html) (accessed on 5 January 2025) [54], AllerTOP v.2.0 (https://www.ddg-pharmfac.net/allertop_test/) (accessed on 5 January 2025) [55], and ToxinPred servers (https://webs.iiitd.edu.in/raghava/toxinpred3/) (accessed on 5 January 2025) [56].

### 4.3. Linear B Cell Epitope Prediction Analysis

The ABCpred server (https://webs.iiitd.edu.in/raghava/abcpred/ABC_submission.html) (accessed on 6 January 2025) utilises artificial neural networks for the prediction of LBL epitopes [57]. The default length of the predicted epitopes is 16 amino acid residues, and the filtering threshold was 0.51. Then, the antigenicity, allergenicity, and toxicity of the LBL epitopes were predicted using the VaxiJen v2.0, AllerTOP v.2.0, and ToxinPred servers, respectively.

### 4.4. Population Coverage Analysis of the Candidate Epitopes

The prevalences of distinct HLA alleles differ across ethnicities. Choosing epitopes that can bind to different HLAs will improve vaccine coverage in endemic populations. In this study, the IEDB (http://tools.iedb.org/population/) (accessed on 6 January 2025) population coverage analysis tool was used to predict the population coverage of selected epitopes [58].

### 4.5. Multi-Epitope Vaccine Construction

β-defensin-3 and PADRE peptide are widely used in vaccine design because they can enhance the immunological effect of vaccines and have a good safety profile [59,60]. The β-defensin-3 adjuvant (GIINTLQKYCRVRGRCAVLSCLPKEEQIGKCSTRGRKCCRRKK) was added to the N-terminus of the vaccine construct. Then, the universal PADRE peptide (AKFVAAWTLKAAA) was linked by the EAAAK linker, a rigid linker that can improve the folding and stability of fusion proteins [61]. The top-ranked LBL, CTL, and HTL epitopes were linked via KK, GGGS, and GPGPG linkers, respectively. The KK linker can improve the water solubility of fusion proteins and reduce protein aggregation, thus reducing the risk of non-specific immunogenicity [62]. GGGS and GPGPG are typical flexible linkers widely used in the design of fusion proteins. It has been shown that these linkers can effectively reduce the steric hindrance between antigenic epitopes by providing flexible junction space while maintaining overall structural stability [63,64]. Lastly, the EGGE linker incorporated the *Yersinia* C-terminal invasion sequence (TAKSKKFPSYTATYQF) at the C-terminus. These linkers enable proper segregation between epitopes and enhance the vaccine’s stability and immunogenicity [33].

### 4.6. Evaluation of Physicochemical Properties of the Multi-Epitope Vaccine

The antigenicity and physicochemical properties of the multi-epitope vaccine were analysed using VaxiJen v2.0 and Expasy ProtParam (https://web.expasy.org/protparam/) (accessed on 9 January 2025) servers [65]. The Protein-Sol server (https://protein-sol.manchester.ac.uk/) (accessed on 9 January 2025) was used to predict the solubility of the vaccine [66].

### 4.7. Prediction and Optimisation of Multi-Epitope Vaccine Structures

The prediction of the vaccine’s secondary structure was carried out via the PSIPRED server (http://bioinf.cs.ucl.ac.uk/psipred/) (accessed on 11 January 2025) [67]. Afterwards, the three-dimensional (3D) spatial structure of the multi-epitope vaccine was modelled using the I-TASSER server (https://zhanggroup.org//I-TASSER/) (accessed on 12 January 2025) [68] and refined using the GalaxyRefine server (https://galaxy.seoklab.org/refine) (accessed on 16 January 2025) [69]. In order to determine the best model, we used validation algorithms ERRAT, VERIFY 3D, and PROCHECK in the SAVES v6.1 server (https://saves.mbi.ucla.edu/) (accessed on 18 January 2025) [70,71,72], as well as the ProSA-Web (https://prosa.services.came.sbg.ac.at/prosa.php) (accessed on 18 January 2025) [73] online tools to validate the refined models.

### 4.8. Prediction of Conformational B-Cell Epitopes

The best model of the vaccine construct was submitted to the ElliPro tool (http://tools.iedb.org/ellipro/) (accessed on 20 January 2025) of the IEDB server to predict the conformational B-cell epitopes with a screening threshold of 0.5 [74].

### 4.9. Molecular Docking

Forming a robust bond between a vaccine and TLRs present on the surface of innate immune cells is essential for initiating potent immune responses [36]. Molecular docking is a good method to assess the ligand–receptor affinity. The PDB files of TLR2 (PDB ID: 6NIG), TLR3 (PDB ID: 1ZIW), and TLR4 (PDB ID: 4G8A) were extracted from the RCSB database (https://www.rcsb.org/) (accessed on 24 January 2025). Then, the best vaccine model and TLR structure files were submitted to the ClusPro 2.0 online server (https://cluspro.bu.edu/login.php) (accessed on 26 January 2025) to perform ligand–receptor docking analysis [75].

### 4.10. Molecular Dynamic Simulations (MDs)

MDs are a valuable tool for assessing the stability of the docking complex of the vaccine construct with TLRs. This study used Gromacs software (v.2020.6) to perform MDs using the Amber 99SB force field. Subsequently, the complex was placed in the centre of a 3D box, 1 nm from the box edge. The Transferable Intermolecular Potential 3P (TIP3P) water model filled the system. Then, Cl^−^ ions were added to make the system electrically neutral and to bring the topological and structural coordinates into a steady state [76]. The steepest descent approach was used to minimize the energy of the solvated system and eradicate three-dimensional conflicts or inappropriate geometries. The solvated system was gradually heated to 300 K for a 100 ps NVT equilibration [36]. The density and pressure of the system were checked by NPT equilibration. The MDs of 100 ns were performed on the system. Finally, the root mean square deviation (RMSD) and the radius of gyration (Rg) were calculated using the gmx rms and gmx gyrate modules, respectively.

### 4.11. Immune Simulation of the Multi-Epitope Vaccine

An immune simulation employing the C-ImmSim server (https://kraken.iac.rm.cnr.it/C-IMMSIM/index.php?page=1) (accessed on 15 February 2025) was performed to assess the vaccine’s potential to stimulate an immuno-protective response in the body [44]. The simulation parameters were 1000 vaccine molecules per injection, three injections at three-week intervals, and the entire immunological simulation was run for 450 days. In this study, the host HLA selection was set to A0101, A0201, B0702, B0801, DRB10101, and DRB1501, and the other simulation parameters were kept as default.

### 4.12. Codon Optimisation and In Silico Cloning

The Java Codon Adaptation Tool (JCat) (http://www.jcat.de/) (accessed on 19 February 2025) [77] was selected to perform reverse translation and codon optimisation and to determine the codon adaptation index (CAI) score and GC percentage of the multi-epitope vaccine construct in *E. coli* K12. Then, the optimized sequence was inserted between the *EcoRI* and *BamHI* restriction enzyme cutting sites of the pET28a (+) expression vector using SnapGene software (version 6.0.2) (https://www.snapgene.com/free-trial/) (accessed on 19 February 2025).

## 5. Conclusions

MPXV and HIV co-infection occurs at a remarkably high rate. This study aimed to develop a novel multi-epitope vaccine against MPXV and HIV using bioinformatics and immunoinformatic methods. We predicted T-cell and B-cell epitopes for the target antigens, and the obtained epitopes and adjuvants were linked via linkers to obtain the final vaccine construct. The vaccine candidate exhibited optimal characteristics such as favourable physicochemical attributes, solubility, potent antigenicity, good immunogenicity, non-toxicity, and non-allergenicity, thus effectively eliciting a strong immune response while avoiding adverse effects. The results of molecular docking and MDs validated the vaccine’s strong stability and affinity for TLRs. Codon optimization yielded favourable CAI values and GC content, indicating good potential for expression in bacterial systems. Therefore, these findings suggest that, with further in vitro and in vivo validation, this candidate vaccine holds promise as an effective vaccine against MPXV and HIV co-infection.

## Figures and Tables

**Table 1:** Selected CTL epitopes for multi-epitope vaccine construction.

Protein	Peptide Sequence	Percentile Rank	Immunogenicity Score	Antigenicity Score	Toxicity	Allergenicity
E8L	SADAAWIIF	0.43	0.4833	0.8244	Non-Toxin	Non-Allergen
A28L	VQHIHFGFR	0.35	0.3831	3.4743	Non-Toxin	Non-Allergen
L1R	VPPIIENRL	0.16	0.3790	0.9944	Non-Toxin	Non-Allergen
Pol	TQDFWEVQL	0.22	0.4021	0.7160	Non-Toxin	Non-Allergen
Nef	SVIGWPTVR	0.03	0.3394	0.7639	Non-Toxin	Non-Allergen
Vpr	FIHFRIGCR	0.38	0.3011	3.1138	Non-Toxin	Non-Allergen

**Table 2:** Selected HTL epitopes for multi-epitope vaccine construction.

Protein	Peptide Sequence	Percentile Rank	Antigenicity Score	IFN-γ Inducer Score	Toxicity	Allergenicity
A29L	KKITNITTKFEQIEK	0.32	0.5631	0.8282	Non-Toxin	Non-Allergen
A35R	HRKVVSSTTQYDHKE	0.24	0.7656	0.5314	Non-Toxin	Non-Allergen
A28L	DSKINIEDDDIIDDD	0.14	0.5307	0.4342	Non-Toxin	Non-Allergen
Pol	VDFRELNKRTQDFWE	0.44	1.0134	0.6691	Non-Toxin	Non-Allergen
Gag	GKKKYKLKHIVWASR	0.47	1.5616	0.5550	Non-Toxin	Non-Allergen
Env	NCSFNISTSIRGKVQ	0.42	0.6658	0.3292	Non-Toxin	Non-Allergen

**Table 3:** Selected LBL epitopes for multi-epitope vaccine construction.

Protein	Peptide Sequence	ABC Pred Score	Antigenicity Score	Toxicity	Allergenicity
A17L	FIRIIRPDYFTFGDTA	0.97	0.5255	Non-Toxin	Non-Allergen
H3L	HEHINDQKFDDVKDNE	0.97	0.5947	Non-Toxin	Non-Allergen
B6R	CDSGYHSLDPNAVCET	0.94	0.7127	Non-Toxin	Non-Allergen
A28L	DDDDDDDDDYNPKPTP	0.91	0.9543	Non-Toxin	Non-Allergen
Gag	HAGPIAPGQMREPRGS	0.93	0.6008	Non-Toxin	Non-Allergen
Vpr	HSRIGVTRQRRARNGA	0.92	1.0919	Non-Toxin	Non-Allergen
Env	SVEINCTRPNNNTRKR	0.92	0.9122	Non-Toxin	Non-Allergen
Vif	PKKIKPPLPSVTKLTE	0.92	0.4677	Non-Toxin	Non-Allergen

## Data Availability

Data are contained within the article and Supplementary Materials.

## Supplementary Materials

The following supporting information can be downloaded at https://www.mdpi.com/article/10.3390/ijms26136313/s1.
