# Supplementary material for: Designing a Multi-Epitope Vaccine Against MPXV and HIV Based on an Immunoinformatic Approach
Source: Int J Mol Sci. 2025 Jun 30;26(13):6313. doi: 10.3390/ijms26136313 (PMC12250479; doi:10.3390/ijms26136313)
Supplement: Supplementary file 1 [file ijms-26-06313-s001.zip › ijms-3690304-supplementary.pdf]

Designing a Multi-Epitope Vaccine Against MPXV and HIV Based on an Immunoinformatic Approach

**Figure S1.**	Theoretical population coverage rates of selected CTL and HTL epitopes. (A) Class I coverage: 98.55% worldwide. (B) Class II coverage: 99.99% worldwide.
**Figure S2.**	Conformational B-cell epitopes present in the vaccine. The light-yellow spheres show epitopes containing (A) 38 residues (AA1-35, AA96-99, and AA101) with 0.881; (B) 49 residues (AA347-349, AA377, AA379-402, and AA 404-424) with 0.723; (C) 59 residues (AA47-56, AA66, AA66-77, AA103, AA103-112, AA114-115, and AA117-146) with 0.715; (D) 81 residues (AA160-174, AA176, AA178-197, AA217, AA219-237, AA239-251, AA253-254, AA264-266, and AA282-289) with 0.633; (E) 3 residues (AA267-269) with 0.535; (F) 5 residues (AA331-335) with 0.520 scores.
**Figure S3.**	Diagram of simulated cloning. In silico cloning of the vaccine construct between the *EcoRI* and *BamHI* restriction sites of the pET-28a (+) vector. The red part represents the vaccine sequence.
**Table S1.**	Theoretical population coverage of areas.
**Table S2.**	Physicochemical properties of the multi-epitope vaccine.
**Table S3.**	Hydrogen bond interactions of the vaccine with TLR2.
**Table S4.**	Hydrogen bond interactions of the vaccine with TLR3.
**Table S5.**	Hydrogen bond interactions of the vaccine with TLR4.

Table S1. Theoretical population coverage of areas.

Population/area	Class I			Class II		
	Coverage[a]	Average hit[b]	pc90[c]	Coverage[a]	Average hit[b]	pc90[c]
World	98.55%	13.79	9.09	99.99%	33.07	24.80
Central Africa	86.04%	9.00	4.30	100.00%	35.58	27.64
Central America	7.76%	0.48	0.65	100.00%	28.14	22.66
East Africa	90.78%	10.06	6.14	100.00%	38.21	30.81
East Asia	98.18%	13.37	8.61	99.90%	26.43	18.65
Europe	99.68%	16.09	12.34	100.00%	34.35	26.42
North Africa	96.03%	11.96	7.43	99.92%	26.09	18.84
North America	99.06%	14.69	10.19	100.00%	38.04	30.98
Northeast Asia	94.70%	10.90	6.89	99.96%	28.82	20.62
Oceania	94.71%	9.98	6.73	100.00%	31.96	24.68
South Africa	93.03%	10.71	6.58	32.10%	1.99	0.88
South America	88.30%	8.76	5.13	100.00%	37.36	30.28
South Asia	94.73%	10.91	6.93	99.99%	31.28	23.67
Southeast Asia	94.56%	10.81	6.86	99.67%	22.06	14.71
Southwest Asia	92.50%	10.36	6.45	99.86%	23.29	16.36
West Africa	95.49%	12.05	7.33	100.00%	34.80	27.06
West Indies	98.98%	15.00	10.50	99.54%	19.54	13.39

a: Projected population coverage. b: Average number of epitope hits/HLA combinations recognized by the population. c: Minimum number of epitope hits/HLA combinations recognized by 90% of the population.

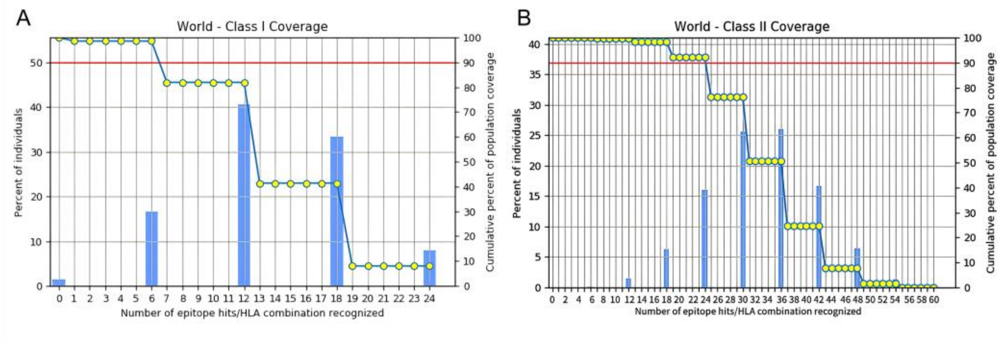

Figure S1. Theoretical population coverage rates of selected CTL and HTL epitopes. (A) Class I coverage: 98.55% worldwide. (B) Class II coverage: 99.99% worldwide. Blue bars indicate the percentage of individuals, and yellow dots indicate the percentage of cumulative population coverage.

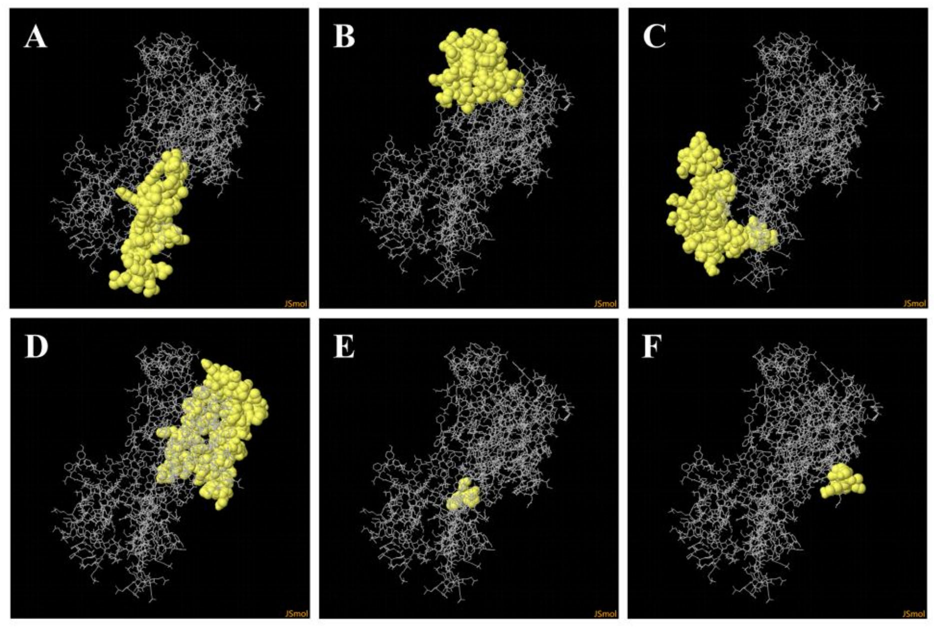

Figure S2. Conformational B-cell epitopes present in the vaccine. The light-yellow spheres show epitopes containing (A) 38 residues (AA1-35, AA96-99, and AA101) with 0.881; (B) 49 residues (AA347-349, AA377, AA379-402, and AA 404-424) with 0.723; (C) 59 residues (AA47-56, AA66, AA66-77, AA103, AA103-112, AA114-115, and AA117-146) with 0.715; (D) 81 residues (AA160-174, AA176, AA178-197, AA217, AA219-237, AA239-251, AA253-254, AA264-266, and AA282-289) with 0.633; (E) 3 residues (AA267-269) with 0.535; (F) 5 residues (AA331-335) with 0.520 scores.

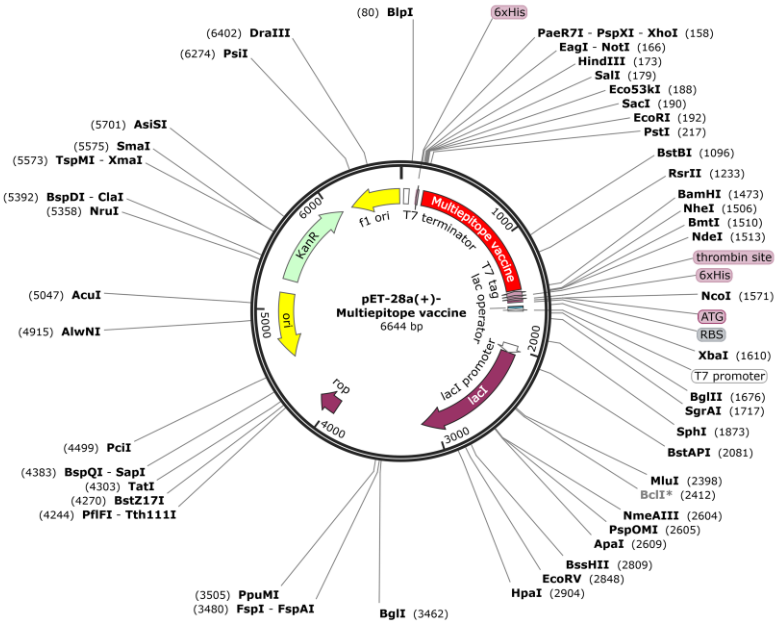

Figure S3. Diagram of simulated cloning. In silico cloning of the vaccine construct between the *EcoRI* and *BamHI* restriction sites of the pET-28a (+) vector. The red part represents the vaccine sequence.

Table S2. Physicochemical properties of the multi-epitope vaccine.

S. no.	Feature	Assessment	Remark
1	Antigenicity	0.7074	Probable antigen
2	Allergenicity	—	Probable non-allergen
3	Toxicity	—	Probable non-toxin
4	Solubility	0.551	Soluble
5	Number of amino acids	425	—
6	Molecular weight	46.63 kDa	—
7	PI	9.73	—
8	Half-life: mammalian reticulocytes, in vitro	30 hours	—
9	Half-life: yeast, in vivo	>20 hours	—
10	Half-life: Escherichia coli, in vivo	>10 hours	—
11	Instability index	36.01	Classifies the protein as
12	Aliphatic index	54.16	Thermostable
13	GRAVY	−0.917	Hydrophilic

Table S3. Hydrogen bond interactions of the vaccine with TLR2.

	**TLR2 residues**						**Vaccine**				
S. no.	Atom no.	Atom name	Residue name	Residue no.	Chain	Bond distance	Atom no.	Atom name	Residue name	Residue no.	Chain
1	64	NZ	LYS	555	A	2.579	70	O	GLY	390	Z
2	116	NZ	LYS	551	A	2.545	33	OD2	ASP	214	Z
3	106	NH1	ARG	547	A	2.838	32	OD1	ASP	214	Z
4	44	NH2	ARG	541	A	2.748	52	OE1	GLN	226	Z
5	43	NH1	ARG	541	A	2.767	52	OE1	GLN	226	Z
6	8	NH2	ARG	521	A	2.977	136	OD1	ASN	244	Z
7	8	NH2	ARG	521	A	2.683	19	O	GLU	243	Z
8	8	NH2	ARG	521	A	2.706	74	OG	SER	212	Z
9	7	NH1	ARG	521	A	2.658	124	O	SER	211	Z
10	5	NE	ARG	521	A	2.926	19	O	GLU	243	Z
11	5	NE	ARG	521	A	2.948	16	OE1	GLU	243	Z
12	25	OD2	ASP	516	A	3.092	146	N	ALA	216	Z
13	79	OD1	ASP	233	C	2.66	29	NZ	LYS	194	Z
14	203	NZ	LYS	168	C	2.554	210	OD1	ASP	128	Z
15	119	NH1	ARG	321	C	2.833	129	NE2	HIS	229	Z
16	219	OD2	ASP	235	C	2.755	29	NZ	LYS	194	Z
17	94	OG1	THR	163	C	2.961	154	O	GLY	267	Z
18	20	OD2	ASP	160	C	2.56	47	NZ	LYS	313	Z
19	20	OD2	ASP	160	C	2.774	101	OG	SER	263	Z
20	19	OD1	ASP	160	C	2.585	47	NZ	LYS	313	Z
21	225	OG1	THR	142	C	2.866	72	NH2	ARG	68	Z
22	38	NZ	LYS	137	C	2.499	166	OG1	THR	270	Z
23	38	NZ	LYS	137	C	2.621	150	O	LYS	154	Z
24	304	OG	SER	117	C	3.101	72	NH2	ARG	68	Z
25	159	OD1	ASN	114	C	2.495	194	NZ	LYS	82	Z
26	87	OD1	ASN	89	C	2.601	139	NZ	LYS	83	Z
27	53	OG1	THR	65	C	2.982	9	NE1	TRP	364	Z
29	35	OE1	GLN	433	D	2.766	26	NH1	ARG	168	Z
30	7	OE2	GLU	383	D	2.521	16	NZ	LYS	190	Z
31	6	OE1	GLU	383	D	2.621	16	NZ	LYS	190	Z

Table S4. Hydrogen bond interactions of the vaccine with TLR3.

	TLR3 Residues						Vaccine				
S. no.	Atom no.	Atom name	Residue name	Residue no.	Chain	Bond distance	Atom no.	Atom name	Residue name	Residue no.	Chain
1	159	ND2	ASN	659	A	2.824	325	O	PRO	387	Z
2	279	NH1	ARG	643	A	2.678	197	O	GLY	388	Z
3	203	ND2	ASN	636	A	2.873	58	OD2	ASP	79	Z
4	116	NH2	ARG	635	A	2.619	58	OD2	ASP	79	Z
5	113	NE	ARG	635	A	2.968	57	OD1	ASP	79	Z
6	468	OG	SER	611	A	2.529	134	NZ	LYS	82	Z
7	308	OE2	GLU	587	A	2.444	134	NZ	LYS	82	Z
8	77	OE2	GLU	533	A	2.578	143	NZ	LYS	313	Z
9	76	OE1	GLU	533	A	2.646	143	NZ	LYS	313	Z
10	269	NH2	ARG	484	A	2.72	107	O	VAL	315	Z
11	315	OD1	ASN	457	A	2.66	18	NZ	LYS	194	Z
12	50	OE2	GLU	434	A	2.681	18	NZ	LYS	194	Z
13	355	O	HIS	432	A	2.767	18	NZ	LYS	194	Z
14	406	OG	SER	385	A	2.799	6	OE1	GLN	226	Z
15	30	OH	TYR	383	A	3.028	6	OE1	GLN	226	Z
16	369	OD1	ASN	380	A	2.475	299	NZ	LYS	190	Z
17	431	OE1	GLU	363	A	3.025	7	NE2	GLN	226	Z

18	65	ND1	HIS	359	A	2.785	242	O	ILE	228	Z
19	362	NZ	LYS	201	A	2.702	150	OD1	ASN	169	Z
20	425	O	LYS	200	A	3.092	151	ND2	ASN	169	Z
21	231	OE1	GLU	175	A	2.837	41	NH2	ARG	285	Z
22	125	OE2	GLU	127	A	2.775	41	NH2	ARG	285	Z
23	343	OG	SER	79	A	2.836	246	OG1	THR	251	Z
24	476	OD2	ASP	36	A	3.165	458	OG	SER	250	Z
25	84	ND1	HIS	32	A	2.674	292	O	HIS	379	Z

Table S5. Hydrogen bond interactions of the vaccine with TLR4.

	**TLR4** Residues						**Vaccine**				
S. no.	Atom no.	Atom name	Residue name	Residue no.	Chain	Bond distance	Atom no.	Atom name	Residue name	Residue no.	Chain
1	71	OE2	GLU	608	A	3.11	93	NE1	TRP	57	Z
2	71	OE2	GLU	608	A	2.523	25	NZ	LYS	50	Z
3	70	OE1	GLU	608	A	2.565	25	NZ	LYS	50	Z
4	46	NH1	ARG	606	A	2.788	289	O	ALA	55	Z
5	46	NH1	ARG	606	A	2.985	38	O	PHE	53	Z
6	105	OE2	GLU	605	A	3.11	85	N	TRP	57	Z
7	242	OE1	GLN	599	A	2.6	263	NZ	LYS	136	Z
8	231	OE1	GLN	578	A	2.634	25	NZ	LYS	50	Z
9	270	OD1	ASN	51	A	2.571	16	NZ	LYS	313	Z
10	120	OD2	ASP	50	A	2.734	16	NZ	LYS	313	Z
11	119	OD1	ASP	50	A	2.561	16	NZ	LYS	313	Z
12	203	N	TYR	46	A	2.806	62	OG	SER	316	Z
13	297	O	ASN	44	A	2.671	62	OG	SER	316	Z
14	9	O	GLU	31	A	2.526	56	NZ	LYS	82	Z
15	6	OE1	GLU	31	A	2.703	200	NZ	LYS	154	Z
16	138	OE1	GLU	27	A	2.653	150	NH2	ARG	272	Z
17	19	NZ	LYS	39	C	2.586	43	OD1	ASP	79	Z
18	64	OE2	GLU	144	C	3.105	93	NE	ARG	68	Z
19	28	NZ	LYS	89	C	2.596	38	O	ASP	73	Z
20	28	NZ	LYS	89	C	2.691	35	OD1	ASP	73	Z
