# Supplementary material for: Designing a Multi-Epitope Vaccine Against MPXV and HIV Based on an Immunoinformatic Approach
Source: Int J Mol Sci. 2025 Jun 30;26(13):6313. doi: 10.3390/ijms26136313 (PMC12250479; doi:10.3390/ijms26136313)
Supplement: Supplementary file 1 [file ijms-26-06313-s001.zip › ijms-3690304-supplementary.doc]

**Designing a Multi-Epitope Vaccine Against MPXV and HIV Based on an Immunoinformatic Approach**

| **Figure S1.** | Theoretical population coverage rates of selected CTL and HTL epitopes. (A) Class I coverage: 98.55% worldwide. (B) Class II coverage: 99.99% worldwide. |
| --- | --- |
| **Figure S2.** | Conformational B-cell epitopes present in the vaccine. The light-yellow spheres show epitopes containing (A) 38 residues (AA1-35, AA96-99, and AA101) with 0.881; (B) 49 residues (AA347-349, AA377, AA379-402, and AA 404-424) with 0.723; (C) 59 residues (AA47-56, AA66, AA66-77, AA103, AA103-112, AA114-115, and AA117-146) with 0.715; (D) 81 residues (AA160-174, AA176, AA178-197, AA217, AA219-237, AA239-251, AA253-254, AA264-266, and AA282-289) with 0.633; (E) 3 residues (AA267-269) with 0.535; (F) 5 residues (AA331-335) with 0.520 scores. |
| **Figure S3.** | Diagram of simulated cloning. In silico cloning of the vaccine construct between the EcoRI and BamHI restriction sites of the pET-28a (+) vector. The red part represents the vaccine sequence. |
| **Table S1.** | Theoretical population coverage of areas. |
| **Table S2.** | Physicochemical properties of the multi-epitope vaccine. |
| **Table S3.** | Hydrogen bond interactions of the vaccine with TLR2. |
| **Table S4.** | Hydrogen bond interactions of the vaccine with TLR3. |
| **Table S5.** | Hydrogen bond interactions of the vaccine with TLR4. |

Table S1. Theoretical population coverage of areas.

| Population/area | Class I | | | Class II | | |
| --- | --- | --- | --- | --- | --- | --- |
|  | Coverage^a^ | Average_hit^b^ | pc90^c^ | Coverage^a^ | Average_hit^b^ | pc90^c^ |
| World | 98.55% | 13.79 | 9.09 | 99.99% | 33.07 | 24.80 |
| Central Africa | 86.04% | 9.00 | 4.30 | 100.00% | 35.58 | 27.64 |
| Central America | 7.76% | 0.48 | 0.65 | 100.00% | 28.14 | 22.66 |
| East Africa | 90.78% | 10.06 | 6.14 | 100.00% | 38.21 | 30.81 |
| East Asia | 98.18% | 13.37 | 8.61 | 99.90% | 26.43 | 18.65 |
| Europe | 99.68% | 16.09 | 12.34 | 100.00% | 34.35 | 26.42 |
| North Africa | 96.03% | 11.96 | 7.43 | 99.92% | 26.09 | 18.84 |
| North America | 99.06% | 14.69 | 10.19 | 100.00% | 38.04 | 30.98 |
| Northeast Asia | 94.70% | 10.90 | 6.89 | 99.96% | 28.82 | 20.62 |
| Oceania | 94.71% | 9.98 | 6.73 | 100.00% | 31.96 | 24.68 |
| South Africa | 93.03% | 10.71 | 6.58 | 32.10% | 1.99 | 0.88 |
| South America | 88.30% | 8.76 | 5.13 | 100.00% | 37.36 | 30.28 |
| South Asia | 94.73% | 10.91 | 6.93 | 99.99% | 31.28 | 23.67 |
| Southeast Asia | 94.56% | 10.81 | 6.86 | 99.67% | 22.06 | 14.71 |
| Southwest Asia | 92.50% | 10.36 | 6.45 | 99.86% | 23.29 | 16.36 |
| West Africa | 95.49% | 12.05 | 7.33 | 100.00% | 34.80 | 27.06 |
| West Indies | 98.98% | 15.00 | 10.50 | 99.54% | 19.54 | 13.39 |

a: Projected population coverage. b: Average number of epitope hits/HLA combinations recognized by the population. c: Minimum number of epitope hits/HLA combinations recognized by 90% of the population.

Figure S1. Theoretical population coverage rates of selected CTL and HTL epitopes. (A) Class I coverage: 98.55% worldwide. (B) Class II coverage: 99.99% worldwide. Blue bars indicate the percentage of individuals, and yellow dots indicate the percentage of cumulative population coverage.

Figure S2. Conformational B-cell epitopes present in the vaccine. The light-yellow spheres show epitopes containing (A) 38 residues (AA1-35, AA96-99, and AA101) with 0.881; (B) 49 residues (AA347-349, AA377, AA379-402, and AA 404-424) with 0.723; (C) 59 residues (AA47-56, AA66, AA66-77, AA103, AA103-112, AA114-115, and AA117-146) with 0.715; (D) 81 residues (AA160-174, AA176, AA178-197, AA217, AA219-237, AA239-251, AA253-254, AA264-266, and AA282-289) with 0.633; (E) 3 residues (AA267-269) with 0.535; (F) 5 residues (AA331-335) with 0.520 scores.

Figure S3. Diagram of simulated cloning. In silico cloning of the vaccine construct between the *EcoRI* and *BamHI* restriction sites of the pET-28a (+) vector. The red part represents the vaccine sequence.

Table S2. Physicochemical properties of the multi-epitope vaccine.

| S. no. | Feature | Assessment | Remark |
| --- | --- | --- | --- |
| 1 | Antigenicity | 0.7074 | Probable antigen |
| 2 | Allergenicity | — | Probable non-allergen |
| 3 | Toxicity | — | Probable non-toxin |
| 4 | Solubility | 0.551 | Soluble |
| 5 | Number of amino acids | 425 | — |
| 6 | Molecular weight | 46.63 kDa | — |
| 7 | PI | 9.73 | — |
| 8 | Half-life: mammalian reticulocytes, in vitro | 30 hours | — |
| 9 | Half-life: yeast, in vivo | >20 hours | — |
| 10 | Half-life: Escherichia coli, in vivo | >10 hours | — |
| 11 | Instability index | 36.01 | Classifies the protein as stable |
| 12 | Aliphatic index | 54.16 | Thermostable |
| 13 | GRAVY | −0.917 | Hydrophilic |

| Table S3. Hydrogen bond interactions of the vaccine with TLR2. | | | | | | | | | | | |
| --- | --- | --- | --- | --- | --- | --- | --- | --- | --- | --- | --- |
|  | **TLR2 residues** | | | | |  | **Vaccine** | | | | |
| S. no. | Atom no. | Atom name | Residue name | Residue no. | Chain | Bond distance | Atom no. | Atom name | Residue name | Residue no. | Chain |
| 1 | 64 | NZ | LYS | 555 | A | 2.579 | 70 | O | GLY | 390 | Z |
| 2 | 116 | NZ | LYS | 551 | A | 2.545 | 33 | OD2 | ASP | 214 | Z |
| 3 | 106 | NH1 | ARG | 547 | A | 2.838 | 32 | OD1 | ASP | 214 | Z |
| 4 | 44 | NH2 | ARG | 541 | A | 2.748 | 52 | OE1 | GLN | 226 | Z |
| 5 | 43 | NH1 | ARG | 541 | A | 2.767 | 52 | OE1 | GLN | 226 | Z |
| 6 | 8 | NH2 | ARG | 521 | A | 2.977 | 136 | OD1 | ASN | 244 | Z |
| 7 | 8 | NH2 | ARG | 521 | A | 2.683 | 19 | O | GLU | 243 | Z |
| 8 | 8 | NH2 | ARG | 521 | A | 2.706 | 74 | OG | SER | 212 | Z |
| 9 | 7 | NH1 | ARG | 521 | A | 2.658 | 124 | O | SER | 211 | Z |
| 10 | 5 | NE | ARG | 521 | A | 2.926 | 19 | O | GLU | 243 | Z |
| 11 | 5 | NE | ARG | 521 | A | 2.948 | 16 | OE1 | GLU | 243 | Z |
| 12 | 25 | OD2 | ASP | 516 | A | 3.092 | 146 | N | ALA | 216 | Z |
| 13 | 79 | OD1 | ASP | 233 | C | 2.66 | 29 | NZ | LYS | 194 | Z |
| 14 | 203 | NZ | LYS | 168 | C | 2.554 | 210 | OD1 | ASP | 128 | Z |
| 15 | 119 | NH1 | ARG | 321 | C | 2.833 | 129 | NE2 | HIS | 229 | Z |
| 16 | 219 | OD2 | ASP | 235 | C | 2.755 | 29 | NZ | LYS | 194 | Z |
| 17 | 94 | OG1 | THR | 163 | C | 2.961 | 154 | O | GLY | 267 | Z |
| 18 | 20 | OD2 | ASP | 160 | C | 2.56 | 47 | NZ | LYS | 313 | Z |
| 19 | 20 | OD2 | ASP | 160 | C | 2.774 | 101 | OG | SER | 263 | Z |
| 20 | 19 | OD1 | ASP | 160 | C | 2.585 | 47 | NZ | LYS | 313 | Z |
| 21 | 225 | OG1 | THR | 142 | C | 2.866 | 72 | NH2 | ARG | 68 | Z |
| 22 | 38 | NZ | LYS | 137 | C | 2.499 | 166 | OG1 | THR | 270 | Z |
| 23 | 38 | NZ | LYS | 137 | C | 2.621 | 150 | O | LYS | 154 | Z |
| 24 | 304 | OG | SER | 117 | C | 3.101 | 72 | NH2 | ARG | 68 | Z |
| 25 | 159 | OD1 | ASN | 114 | C | 2.495 | 194 | NZ | LYS | 82 | Z |
| 26 | 87 | OD1 | ASN | 89 | C | 2.601 | 139 | NZ | LYS | 83 | Z |
| 27 | 53 | OG1 | THR | 65 | C | 2.982 | 9 | NE1 | TRP | 364 | Z |
| 29 | 35 | OE1 | GLN | 433 | D | 2.766 | 26 | NH1 | ARG | 168 | Z |
| 30 | 7 | OE2 | GLU | 383 | D | 2.521 | 16 | NZ | LYS | 190 | Z |
| 31 | 6 | OE1 | GLU | 383 | D | 2.621 | 16 | NZ | LYS | 190 | Z |

| Table S4. Hydrogen bond interactions of the vaccine with TLR3. | | | | | | | | | | | |
| --- | --- | --- | --- | --- | --- | --- | --- | --- | --- | --- | --- |
|  | TLR3 Residues | | | | |  | Vaccine | | | | |
| S. no. | Atom no. | Atom name | Residue name | Residue no. | Chain | Bond distance | Atom no. | Atom name | Residue name | Residue no. | Chain |
| 1 | 159 | ND2 | ASN | 659 | A | 2.824 | 325 | O | PRO | 387 | Z |
| 2 | 279 | NH1 | ARG | 643 | A | 2.678 | 197 | O | GLY | 388 | Z |
| 3 | 203 | ND2 | ASN | 636 | A | 2.873 | 58 | OD2 | ASP | 79 | Z |
| 4 | 116 | NH2 | ARG | 635 | A | 2.619 | 58 | OD2 | ASP | 79 | Z |
| 5 | 113 | NE | ARG | 635 | A | 2.968 | 57 | OD1 | ASP | 79 | Z |
| 6 | 468 | OG | SER | 611 | A | 2.529 | 134 | NZ | LYS | 82 | Z |
| 7 | 308 | OE2 | GLU | 587 | A | 2.444 | 134 | NZ | LYS | 82 | Z |
| 8 | 77 | OE2 | GLU | 533 | A | 2.578 | 143 | NZ | LYS | 313 | Z |
| 9 | 76 | OE1 | GLU | 533 | A | 2.646 | 143 | NZ | LYS | 313 | Z |
| 10 | 269 | NH2 | ARG | 484 | A | 2.72 | 107 | O | VAL | 315 | Z |
| 11 | 315 | OD1 | ASN | 457 | A | 2.66 | 18 | NZ | LYS | 194 | Z |
| 12 | 50 | OE2 | GLU | 434 | A | 2.681 | 18 | NZ | LYS | 194 | Z |
| 13 | 355 | O | HIS | 432 | A | 2.767 | 18 | NZ | LYS | 194 | Z |
| 14 | 406 | OG | SER | 385 | A | 2.799 | 6 | OE1 | GLN | 226 | Z |
| 15 | 30 | OH | TYR | 383 | A | 3.028 | 6 | OE1 | GLN | 226 | Z |
| 16 | 369 | OD1 | ASN | 380 | A | 2.475 | 299 | NZ | LYS | 190 | Z |
| 17 | 431 | OE1 | GLU | 363 | A | 3.025 | 7 | NE2 | GLN | 226 | Z |
| 18 | 65 | ND1 | HIS | 359 | A | 2.785 | 242 | O | ILE | 228 | Z |
| 19 | 362 | NZ | LYS | 201 | A | 2.702 | 150 | OD1 | ASN | 169 | Z |
| 20 | 425 | O | LYS | 200 | A | 3.092 | 151 | ND2 | ASN | 169 | Z |
| 21 | 231 | OE1 | GLU | 175 | A | 2.837 | 41 | NH2 | ARG | 285 | Z |
| 22 | 125 | OE2 | GLU | 127 | A | 2.775 | 41 | NH2 | ARG | 285 | Z |
| 23 | 343 | OG | SER | 79 | A | 2.836 | 246 | OG1 | THR | 251 | Z |
| 24 | 476 | OD2 | ASP | 36 | A | 3.165 | 458 | OG | SER | 250 | Z |
| 25 | 84 | ND1 | HIS | 32 | A | 2.674 | 292 | O | HIS | 379 | Z |

| Table S5. Hydrogen bond interactions of the vaccine with TLR4. | | | | | | | | | | | |
| --- | --- | --- | --- | --- | --- | --- | --- | --- | --- | --- | --- |
|  | **TLR4** Residues | | | | |  | **Vaccine** | | | | |
| S. no. | Atom no. | Atom name | Residue name | Residue no. | Chain | Bond distance | Atom no. | Atom name | Residue name | Residue no. | Chain |
| 1 | 71 | OE2 | GLU | 608 | A | 3.11 | 93 | NE1 | TRP | 57 | Z |
| 2 | 71 | OE2 | GLU | 608 | A | 2.523 | 25 | NZ | LYS | 50 | Z |
| 3 | 70 | OE1 | GLU | 608 | A | 2.565 | 25 | NZ | LYS | 50 | Z |
| 4 | 46 | NH1 | ARG | 606 | A | 2.788 | 289 | O | ALA | 55 | Z |
| 5 | 46 | NH1 | ARG | 606 | A | 2.985 | 38 | O | PHE | 53 | Z |
| 6 | 105 | OE2 | GLU | 605 | A | 3.11 | 85 | N | TRP | 57 | Z |
| 7 | 242 | OE1 | GLN | 599 | A | 2.6 | 263 | NZ | LYS | 136 | Z |
| 8 | 231 | OE1 | GLN | 578 | A | 2.634 | 25 | NZ | LYS | 50 | Z |
| 9 | 270 | OD1 | ASN | 51 | A | 2.571 | 16 | NZ | LYS | 313 | Z |
| 10 | 120 | OD2 | ASP | 50 | A | 2.734 | 16 | NZ | LYS | 313 | Z |
| 11 | 119 | OD1 | ASP | 50 | A | 2.561 | 16 | NZ | LYS | 313 | Z |
| 12 | 203 | N | TYR | 46 | A | 2.806 | 62 | OG | SER | 316 | Z |
| 13 | 297 | O | ASN | 44 | A | 2.671 | 62 | OG | SER | 316 | Z |
| 14 | 9 | O | GLU | 31 | A | 2.526 | 56 | NZ | LYS | 82 | Z |
| 15 | 6 | OE1 | GLU | 31 | A | 2.703 | 200 | NZ | LYS | 154 | Z |
| 16 | 138 | OE1 | GLU | 27 | A | 2.653 | 150 | NH2 | ARG | 272 | Z |
| 17 | 19 | NZ | LYS | 39 | C | 2.586 | 43 | OD1 | ASP | 79 | Z |
| 18 | 64 | OE2 | GLU | 144 | C | 3.105 | 93 | NE | ARG | 68 | Z |
| 19 | 28 | NZ | LYS | 89 | C | 2.596 | 38 | O | ASP | 73 | Z |
| 20 | 28 | NZ | LYS | 89 | C | 2.691 | 35 | OD1 | ASP | 73 | Z |
